# The Wnt signaling cascade in the pathogenesis of osteoarthritis and related promising treatment strategies 

**DOI:** 10.3389/fphys.2022.954454

**Published:** 2022-09-02

**Authors:** Jinchao Cheng, Min Li, Ruijun Bai

**Affiliations:** ^1^ Department of Orthopaedics, Xuancheng Central Hospital, Xuancheng, China; ^2^ Department of Orthopaedics, Xiangya Hospital, Central South University, Changsha, China

**Keywords:** chondrocyte, wnt signaling, osteoarthritis, *β*-catenin, therapy

## Abstract

Osteoarthritis (OA) is the most prevalent joint disease, characterized by the degradation of articular cartilage, synovial inflammation, and changes in periarticular and subchondral bone. Recent studies have reported that Wnt signaling cascades play an important role in the development, growth, and homeostasis of joints. The Wnt signaling cascade should be tightly regulated to maintain the homeostasis of cartilage in either the over-activation or the suppression of Wnt/*β*-catenin, as this could lead to OA. This review summarizes the role and mechanism of canonical Wnt cascade and noncanonical Wnt cascade experiments *in vivo* and *in vitro*. The Wnt cascade is controlled by several agonists and antagonists in the extracellular medium and the cytoplasm. These antagonists and agonists serve as key molecules in drug intervention into the Wnt pathway and may provide potential approaches for the treatment of OA. However, the complexity of the Wnt signaling cascade and the pharmaceutical effects on its mechanism are still not fully understood, which forces us to conduct further research and develop efficient therapeutic approaches to treat OA.

## Introduction

Osteoarthritis (OA) is the most prevalent joint disease, characterized by breakdown of the articular cartilage, abnormal bone remodeling, and osteophytosis that lead to chronic pain and functional restrictions of the affected joints. Radiographic evidence of OA is found in the majority of people by 65 years old, and approximately 80% of people aged over 75 years old are affected (Arden and Nevitt, 2006). A large number of studies have reported the incidence of OA, but its pathogenesis has not been fully elucidated.

Articular cartilage degeneration is a major cause of pathological changes in OA. Cartilage is an avascular, neural, lymphatic, and viscoelastic connective tissue, and its main function is to bear loads, promoting the frictionless movement of the joints (Kuettner et al., 1991). Chondrocytes, the only cell population in adult articular cartilage, are capable of responding to structural changes, including the synthesis of collagen and the degeneration of the extracellular matrix (ECM), although the capacity of adult articular chondrocytes to regenerate normal cartilage matrix architecture is limited and declines with age (Loeser, 2009; Tian et al., 2015). Collagens, mainly type II collagen and aggrecan, are the main components of articular cartilage (Roughley, 2001; Lambert et al., 2019). The degeneration of articular cartilage is thought to be due to a disturbance of the balance between the synthesis and metabolism activity, including decreasing the synthesis of type II collagen and proteoglycan and increasing the synthesis of matrix metalloproteinases (MMPs), thrombospondin motifs, and inflammatory factor (van der Kraan and van den Berg, 2000; Davidson et al., 2006; van der Kraan and van den Berg, 2012; Wiegertjes et al., 2019).

### Structure and function of Wnt protein

Wnt signaling cascades are among the most critical biological pathways and are involved in several processes, including joint formation during embryonic skeleton genesis and joint homeostasis and disease in postnatal life (Ladher et al., 2000; Hartmann, 2007; Clevers and Nusse, 2012). Wnts are proteins that have a molecular weight of 36–49 kDa, and their N-terminal peptide contains 350–400 amino acids (Sassi et al., 2014a). At present, Wnt family consists of at least 19 known members in mammals that bind to different intracellular receptors and activate several downstream pathways (Lories et al., 2013; Sassi et al., 2014a). Among the Wnt proteins, the function of Wnt3a was the first to be defined (Willert et al., 2003).

The activation of a Wnt pathway requires the interaction of Wnt proteins with two receptors, one from the Fzl family and the other from the lipoprotein receptor-related protein (LRP) family. The Fzl receptors are generally grouped into seven types belonging to the G-protein–receptor family, and the N-termini of Fzls are extracellular and rich in cysteine, which can interact directly with Wnt protein (Bhanot et al., 1996; Yang-Snyder et al., 1996; Foord et al., 2005). LRP is a transmembrane protein and is considered a coreceptor protein of the Wnt pathway that can transduce canonical Wnt signals *via* interactions with axin (Mao et al., 2001; Barik et al., 2014).

Several proteins are involved in the Wnt cascade in the cytoplasm. Axin interacts with the C-terminal region of the coreceptor LRP and it antagonizes the intracellular level of Wnt signaling (Zeng et al., 1997; Staal and Clevers, 2000). Adenomatous polyposis *coli* (APC) is another protein in the cytoplasm, which is combined with axin, disheveled (Dvl), and casein kinase I (CK-I) to form a degeneration complex. It plays an important role in maintaining the balance of *β*-catenin by degrading excess *β*-catenin (Munemitsu et al., 1995; Behrens et al., 1998; Staal and Clevers, 2000). *β*-Catenin is a key mediator for the Wnt canonical cascade and plays a critical role in cell adhesion and interactions among cells (Lories et al., 2013).

### The canonical cascade and noncanonical cascades

**FIGURE 1:**
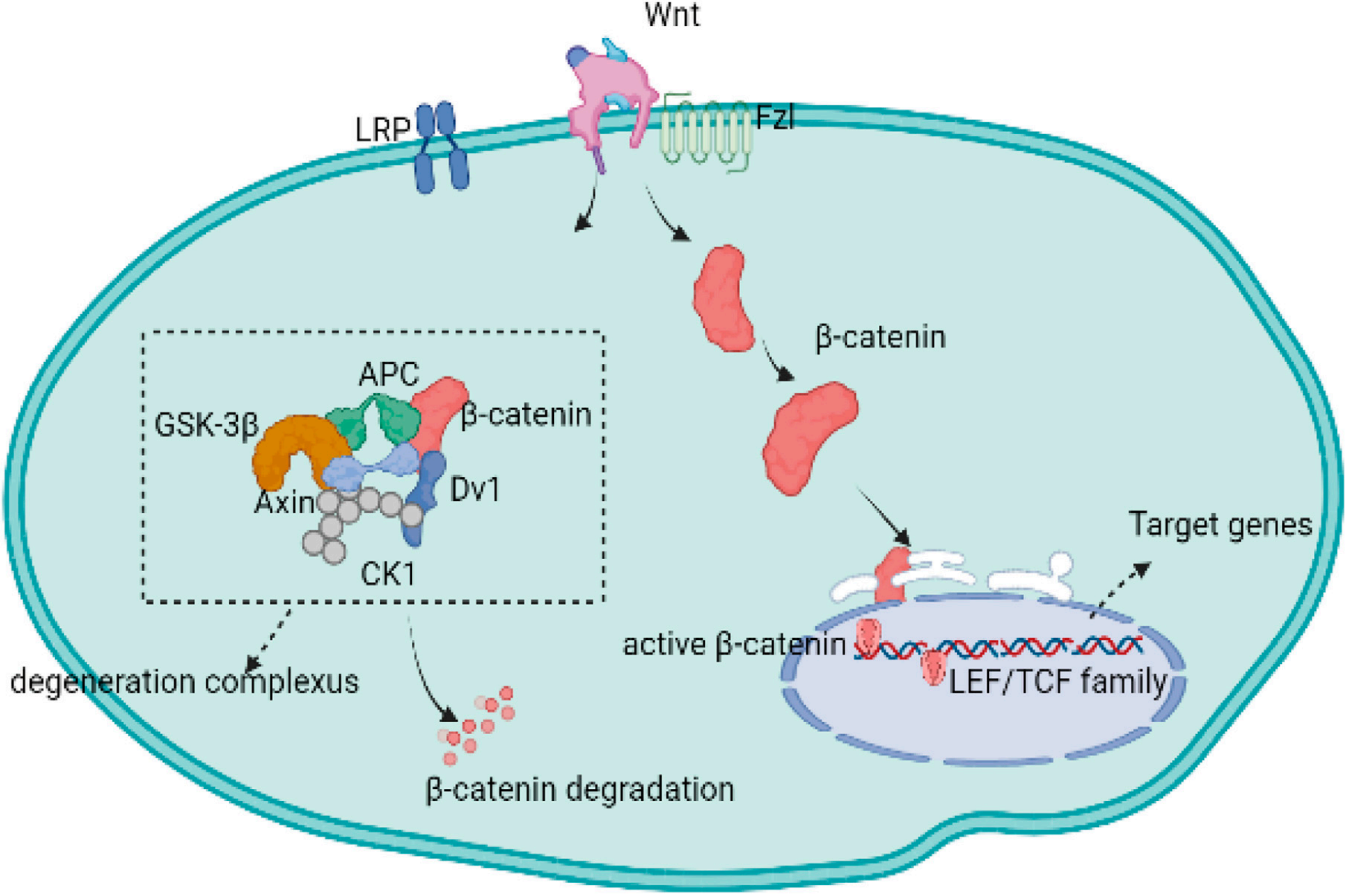
The canonical Wnt signaling pathway is dependent on the activation of intracellular molecule *β*-catenin. In the absence of Wnt-binding Fz receptors, *β*-catenin is isolated into a protein, phosphorylated, and subsequently degraded by the destruction complex. Upon binding to the Fzl receptors and LRP5/6 co-receptors, the Wnt/β-catenin signaling pathways is activated while the degradation complex of *β*-catenin is inhibited, allowing the newly synthesized *β*-catenin to accumulate in the cytoplasm and transfer to the nucleus. Nuclear *β*-catenin can replace the transcription corepressors in TCF transcription factors and promote the activation of gene transcription programs.

The canonical cascade is dependent on the activation of *β*-catenin. The Wnt proteins, the Fzl receptor family, and LRP form a complex that activates the downstream canonical *β*-catenin pathway. Moreover, Wnt-receptor complexes are mediated and controlled by downstream degradation complexes composed of APC, axin, and glycogen synthase kinase-3 *β* (GSK-3β) (Behrens et al., 1998; Staal and Clevers, 2000; Li et al., 2012). Once a Wnt protein binds to the Fzl receptor and the LRP protein, the degeneration complex is inhibited in the cytoplasm, while the complex formed by Wnt and its receptors binds to *β*-catenin, allowing *β*-catenin to accumulate in the cytoplasm and translocate to the nucleus (Rao and Kuhl, 2010; van den Bosch et al., 2016; De Santis et al., 2018). After translocation, *β*-catenin interacts with transcription factors from the LEF/TCF family, which is composed of TCF-1, LEF-1, TCF-3, and TCF-4 (Clevers and Nusse, 2012; Yu and Virshup, 2014; Bengoa-Vergniory and Kypta, 2015). Then, *β*-catenin in the nucleus blocks transcription of the target genes. If the Wnt protein does not bind to the Fzl receptor or the LRP protein, the excess *β*-catenin proteins are degraded by the destruction complex in the cytoplasm (Figure 1) (Johnson and Kamel, 2007).

The noncanonical signaling pathways do not depend on the activation of *β*-catenin. Although the noncanonical Wnt signaling pathway plays an important role in the regulation of cell shape, adhesion, transfer, differentiation, and communication, little research has been reported on these pathways (Sugimura and Li, 2010; Wallingford and Mitchell, 2011; Clark et al., 2012). Under some conditions, it may even lead to the suppression of *β*-catenin (Ishitani et al., 2003). The noncanonical cascade is largely regulated by several extracellular and intracellular signaling molecules, including G-protein-coupled receptors, c-Jun N-terminal kinase (JNK), p38, and triphosphate (IP3)-intercellular calcium (Sugimura and Li, 2010). The receptors involved in the noncanonical cascade mainly include Frizzled, receptor tyrosine kinase (Ryk), and tyrosine-protein kinase transmembrane receptor (Ror2) (Usami et al., 2016).

**FIGURE 2:**
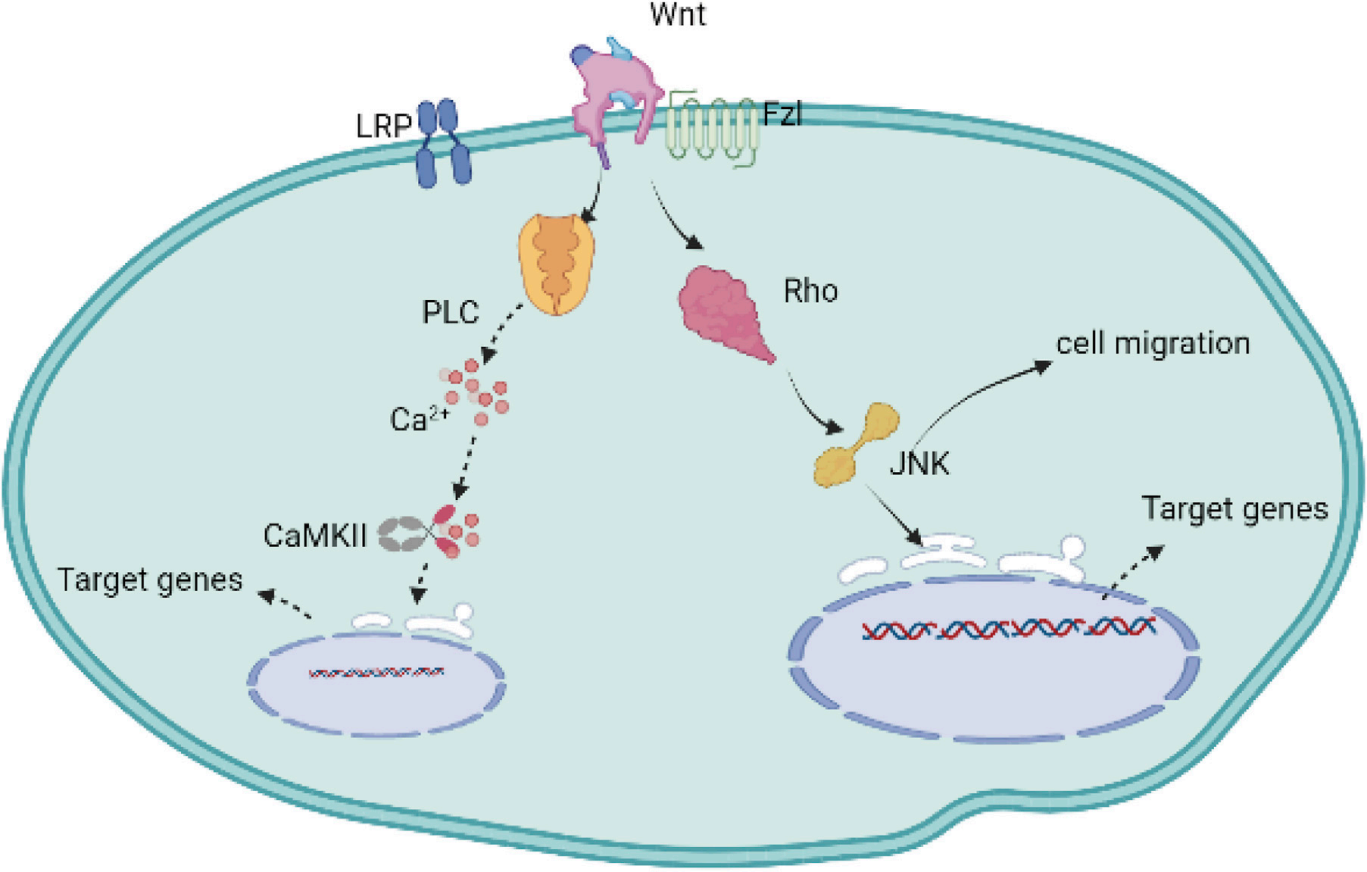
The non-canonical Wnt signaling cascade is activated without the involvement and activation of *β*-catenin. The Wnt protein binds to Fzl and other ligand receptors without the engagement of the LRP co-receptors. One of these cascades is triggered by the stimulation of the intracellular Ca^2+^ secondary to the production of the cytoplasmic PLC, subsequently activating the transcription factors in cell nucleus through the regulation of CaMKII; the other non-canonical Wnt signaling cascade relies on the increase of Rho following the activation of mitogen-activated kinases such as c-Jun N-terminal kinase (JNK), targeting the genes, and regulating cell proliferation and migration.

The noncanonical cascade includes two types of signaling pathways, grouped according to the molecules regulated in the pathways. One cascade is WNT/PCP through Rho and JNK (c-jun N-terminal kinase), leading to the activation of downstream kinases, such as mitogen-activated kinases, including c-Jun, N-terminal kinase (JNK), and protein kinase, which play an important role in cell migration; the other cascade is WNT/Ca^2+^, through protein kinase C (PKC) and calcium/calmodulin-dependent kinase II (CaMK II), resulting in intracellular calcium release (Figure 2) (Liu et al., 2008; Sugimura and Li, 2010; Teo and Kahn, 2010; van Amerongen, 2012).

### Role of Wnts in OA

A large number of studies have studied the role of Wnt in OA; even so, the mechanisms of Wnt protein have not been fully understood. The function of Wnts in OA is reflected in its ability to affect bone formation, endochondral ossification, bone growth and repair, and joint development (Lories et al., 2013). Bone growth requires chondrocytes to go through a complex process, including proliferation, migration, condensation, and adherence. During this process, chondrogenic differentiation passes through different stages in which the skeleton “model” is laid down, and the cells proliferate and become further differentiated *via* hypertrophy (Lefebvre and Bhattaram, 2010; Lories et al., 2013). Wnt3a can promote chondrocyte hypertrophy and differentiation through the canonical Wnt cascade, and suppression of the expression of Wnt3a could inhibit chondrogenic hypertrophy and EXT1 gene expression; furthermore, the Wnt/β-catenin pathway can regulate the expression of the EXT1 gene (Wang et al., 2019). Studies have indicated that Wnt5a and Wnt5b can induce chondrogenesis to affect chondrocyte proliferation and differentiation through the expression of the cytokines cyclinD1 and p130 (Ladher et al., 2000; Yang et al., 2003; Ge et al., 2017). Church et al. (2002) found that Wnt5a and Wnt5b promote early chondrogenesis through the activation of the Wnt noncanonical signaling pathway. Huang et al. (Shi et al., 2016; Huang et al., 2017) found that Wnt5a could activate the Wnt/β-catenin pathway, which leads to increased release of inflammatory mediators, aggravated cartilage damage and inflammatory responses, and accelerated OA process. The Wnt/β-catenin pathway also affects the activities of osteoblasts and osteoclasts, which play an important role in bone growth and remodeling. The osteocytes can produce Wnt protein and stimulate osteocyte maturation; however, they also produce sclerostin, which competitively antagonizes Wnt protein and binds to the LRP receptor (Li et al., 2005; Robling et al., 2008). Wnt proteins such as Wnt7b, Wnt-receptor LRP, and Wnt antagonist make significant contributions to activating the Wnt pathways and regulating osteocyte maturation and bone growth (Li et al., 2005; Robling et al., 2008).

A previous study also found that Wnt7b is closely related to inflammation in articular cartilage, bone, and synovial tissues derived from OA and RA patients (Nakamura et al., 2005). Wnt7a also plays an important role in the pathological process of OA; it could inhibit IL-1β-induced catabolic gene expression and prevent articular cartilage damage in experimental osteoarthritis. Meanwhile, Wnt7a decreased during human chondrocyte dedifferentiation with both no treatment and IL-1 treatment *in vitro,* while Wnt5a presented the opposite result against Wnt7a *in vitro* (Sassi et al., 2014b; Gibson et al., 2017).

Although a series of studies reported that most Wnts could activate the Wnt cascade and noncascade pathway, resulting in cartilage degeneration, Wnt16 could activate Wnt signaling and prevent the exacerbation of cartilage breakdown through exaggerated Wnt activation (Nalesso et al., 2017; Kovacs et al., 2019). Tong et al. (Nalesso et al., 2017; Tong et al., 2019) found that Wnt16 overexpression in chondrocytes in mice significantly inhibits chondrocyte hypertrophy in skeletal development and that Wnt16 activates the PCP/JNK pathway to inhibit chondrocyte hypertrophy. Hua et al. (2022) showed that Wnt16 is a weak activator of *β*-catenin and that upregulating Wnt16 levels could reduce disease progression through the Wnt/β-catenin pathway in temporomandibular OA.

**FIGURE 3:**
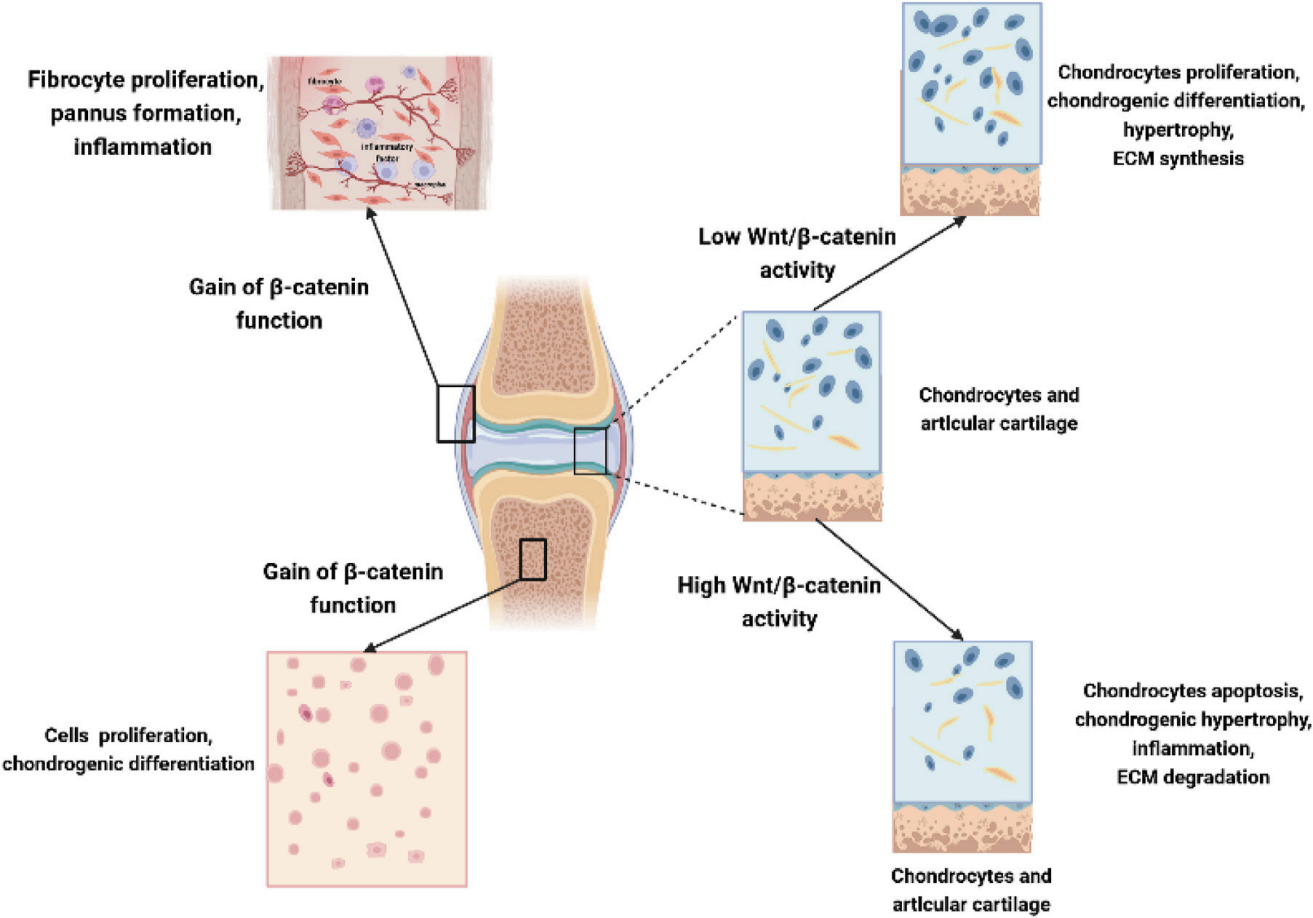
Complex role of Wnts in chondrocytes, synoviocytes, and mesenchymal stem cells. Low Wnt/β-catenin activity in chondrocytes promotes chondrocyte proliferation, chondrogenic differentiation, chondrocyte hypertrophy, and increased ECM synthesis, while high Wnt/β-catenin activity leads to chondrocyte apoptosis, hypertrophy, increased inflammation, and degradation of ECM in joints. Overexpression *β*-catenin contributes to the fibrocyte proliferation, pannus formation, and local inflammation in synoviocytes; moreover, it could also promote the proliferation and chondrogenic differentiation of MSCs in joints.

Due to the large number of studies devoted to investigating the role of Wnt molecules and their influence in OA, our understanding of Wnt biology has rapidly increased, as summarized in Table 1. Although several studies have emphasized the important role of Wnt signaling in bone and joint development, numerous studies have also suggested that the inhibition of Wnts and the Wnt-related pathway is beneficial for OA. Due to the complexity and diversity of Wnt signaling in different cells in joints, abnormally high Wnt activity or low expression of the Wnt signaling pathway in OA may lead to cartilage damage and ultimately accelerate OA (Figure 3). It is necessary and important to maintain balanced biological activity of the Wnt-related pathway due to the complexity of these communication systems in OA.

**TABLE 1:** Role of Wnt in OA.

Protein	References	Role of joints
Wnt3a	Wang X et al. (2019)	Promoting the chondrocyte hypertrophy and differentiation
Wnt4	Fan Liying et al. (2018)	Up-regulated in the progress of differentiation in ATDC5 via Wnt-4/β-catenin signaling
	Nan Yao et al. (2021)	Downregulating mRNA and protein expression of Wnt-4/β-catenin reduced pathological damage and matrix degradation of articular cartilage in KOA rats
Wnt5a	Xianpeng Ge et al. (2017)	Activating Wnt5a facilitated chondrocyte proliferation, hypertrophy, and migration
	Huang G et al. (2107)	Upregulated in OA and inducing catabolic signaling and MMP production in human articular chondrocytes
Wnt5b	Yingzi Yang et al. (2003)	Promoting the chondrocyte hypertrophy, proliferation, and differentiation
Wnt6	Arjen B Blom et al. (2009)	Upregulated in the synovium during the early phase of mouse collagenase-induced OA.
Wnt7a	Gibson Averi L et al. (2017)	Wnt7a protected the cartilage damaged through promoting joint cartilage integrity, inhibiting inflammatory stimuli-induced catabolic gene expression and the activities of MMP activity in joints
	Sassi N et al. (2014)	Down-regulated in chondrocytes during the progress of human chondrocyte de-differentiation
Wnt7b	Xiaofeng Li et al. (2005)	Regulating the osteocyte maturation and bone growth
	Ma B et al. (2012)	Overexpression induced by IL-1 and accompanied with the expression of MMP-1, MMP-3, and MMP-13 via canonical Wnt-7b/β-catenin in human chondrocytes
Wnt8a	Carmen García-Ibarbia et al. (2013)	Downregulated of Wnt8a activity is related with hip fractures in patients compared to those with osteoarthritis
Wnt8b	Florian Witte et al. (2009)	Unknown
Wnt9a	Xuan Fengjun et al. (2019)	Wnt9a was highly expressed in superficial zone cells of articular cartilage and regulated the expression of Prg4 *via* Wnt/β-catenin signaling pathway
Wnt10a	Huang Junjie et al. (2020)	Wnt10a increased the expression of inflammatory cytokines in OA patient SMSCs, whereas Wnt10a had a mild protective effect on cartilage integrity in a rat model
Wnt10b	Kazushi Imai et al. (2006)	Wnt10b was detected in RA and OA synovium. The expression was parallel with the degree of inflammatory cell infiltration and tissue fibrosis
Wnt11	Michael S Friedman et al. (2009)	Wnt11 promotes osteoblast maturation and mineralization by activating Rspo2 expression through Wnt/β-catenin signaling pathway
Wnt16	Tong W et al. (2019)	Wnt16 overexpression in chondrocytes of mice significantly inhibited chondrocyte hypertrophy during skeletal development through the activation of non-canonical signaling pathways
	Francesco Dell’accio et al. (2008)	Wnt-16 and *β*-catenin were dramatically upregulated in areas of the same joint with moderate to severe OA damage

### The role of exosomes in Wnt signaling in OA

In recent years, exosomes are increasingly reported as part of a therapeutic strategy in OA and as having a role in regulating the renewal and regenerative capabilities of chondrocytes. Exosomes are derived from a variety of cell types with 30–100 nm cup-shaped vesicles in diameter (Wu et al., 2020). Exosomes are released from protocells by fusion with the plasma membrane, and they are mainly composed of coding and noncoding RNAs, proteins, antigen-presenting molecules, and DNA (Zhou et al., 2020; Moghiman et al., 2021). Because of their size and composition, exosomes are important therapeutic agents for many diseases and are useful materials for tissue engineering. At present, exosomes are thought to be among the most important factors regulating inflammatory activities in many diseases, such as OA.

OA is influenced by multiple cell types, including chondrocytes and synovial fibroblasts, and both of these cells are regulated by the Wnt signaling pathways and have the ability to secrete exosomes and regulate cartilage metabolism (Kato et al., 2014; Chen et al., 2018). Several researchers have found that exosomes derived from a variety of cells could influence the progression of OA through the Wnt signaling pathway (Table 2). A recent study found that exosomes derived from platelet-rich plasma could promote chondrocyte proliferation and migration and decrease OA chondrocyte apoptosis by inhibiting the expression of Wnt5a/β-catenin in IL-1β-treated chondrocytes (Liu et al., 2019). Zhao et al. (2020) also indicated the role of exosomes in suppressing inflammation by decreasing the proinflammatory markers IL-6, NF-κB, and tumor necrosis factor alpha (TNF-α) and by upregulating the anti-inflammatory cytokine IL-10 level by activating *β*-catenin. Moreover, animal experiments indicated that exosomes could inhibit the progression of early OA and prevent severe damage to articular cartilage by targeting the Wnt5a gene and promoting chondrocyte proliferation and migration *in vitro* (Mao et al., 2018). However, other scholars found that exosomes extracted from synovial mesenchymal stem cells go beyond contributing to the proliferation and migration of chondrocytes by causing the side effect of degrading the cartilage matrix. The Wnt molecules Wnt5a and Wnt5b target the downstream protein of YAP/TAZ and are responsible for this process through noncanonical Wnt cascades (Tao et al., 2017). These studies indicate that the Wnt signaling pathway is an important regulatory mechanism for exosomes in decreasing inflammatory activity and chondrocyte apoptosis, as well as promoting cell proliferation and alleviating articular cartilage damage in OA.

**TABLE 2:** | Role of the exosome and Wnt in OA.

Author	Subjects	Source of exosomes	Path involved	Main results
Liu Xuchang et al. (2019)	New Zealand rabbits OA model and chondrocytes	Platelet-rich plasma	Wnt5a/β-catenin	Promoting the chondrocyte proliferation, migration, and decreasing OA chondrocytes apoptosis through inhibiting the expression of β-catenin, Wnt5a in IL-1β-treated chondrocytes
Dong Jisheng et al. (2021)	Chondrocytes of Sprague–Dawley rats	BM-MSCs	β-Catenin	Exosomes derived from BM-MSCs restricted the IL-1β-induced chondrocytes damage through inhibit the activation of Wnt/β-catenin pathway
Chen Zhao et al. (2020)	Patient-derived chondrocytes	Adipose-derived stem cells	β-Catenin	Exosomes downregulated the expression of pro-inflammatory markers IL-6 and TNF-α, upregulated the anti-inflammatory cytokine IL-10 expression, and protected articular chondrocytes from apoptosis *via* activating the β-catenin
Tao Shi-Cong et al. (2017)	Patient-derived chondrocytes	Synovial mesenchymal stem cells	Wnt5a/5b-YAP/TAZ	Exosomes promote the proliferation and migration of chondrocytes with the side effect of decreasing ECM secretion through *via* the Wnt signaling pathway induced by Wnt5a and Wnt5b
Mao Guping et al. (2018)	Patient-derived chondrocytes and C57B/L10 mice with a collagenase-induced OA model	Human mesenchymal stem	Wnt5a	Exosomes promote the chondrocyte proliferation, migration, and reduced cartilage matrix synthesis by enhancing the expression of Wnt5a. Animal experiment also indicated exosomes inhibited the progression of early OA and prevented the severe damage to articular cartilage
Zhu Chunhui et al. (2021)	OA patient cartilage and CHON-001 cells	CHON-001 cells	Wnt5b	Exosome-mediated transfer of circ_0001846 modulated IL-1β-induced chondrocytes damage by targeting the expression of Wnt5b

### Role of miRNAs in the Wnt signaling pathway in OA

MicroRNAs (miRNAs) are small noncoding RNAs with the capability of modulating essential biological processes in animals and plants. Since they were first discovered in *Caenorhabditis elegans* in 1993, miRNAs have been found to participate in regulating posttranscriptional gene expression, either by inhibiting messenger RNA (mRNA) translation or by promoting mRNA degradation (Saliminejad et al., 2019). More importantly, the abnormal expression of miRNAs could affect the pathological process of various diseases, such as OA, due to their role in regulating and modulating gene expression, which is related to the various physiological and pathological mechanisms in chondrocytes.

In recent decades, miRNA levels, which are posttranscriptionally related, have been extensively studied in OA, and the results show that miRNAs have both protective and destructive effects on articular cartilage in OA. MicroRNAs influence multiple intracellular signaling pathways to alter chondrocyte status, and the Wnt pathway is one of the most important signaling pathways that are targeted by miRNAs in OA. In general, miRNAs can activate or suppress the Wnt signaling pathway by targeting its components, while Wnt signaling also negatively influences the expression of miRNAs. For these reasons, miRNAs and Wnt signaling pathways are especially important for maintaining cartilage metabolism, joint inflammation, chondrocyte proliferation, differentiation, and apoptosis in OA, as presented in Table 3.

**TABLE 3:** miRNAs targeting the Wnt signaling pathway in the pathogenesis of OA.

Name	References	Target	Main results
miR-1	Yang Yang et al. (2021)	FZD7	Decrease the expression of catabolic genes
miR-127-3p	Dong Jisheng et al. (2021)	β-Catenin	Decrease the expression of MMP-13, ADAMTS-5, TNF-α, and IL-6
miR-195-5p	Yang Shu et al. (2019)	β-Catenin	Increase the expression of TNF-α, IL-6, and IL-1β in chondrocytes and induce the chondrocytes apoptosis
miR-27a/b	Sara Cheleschi et al. (2017)	β-Catenin	Downregulate the expression of MMP-13, ADAMTS-5, and HDAC-4
miR-140	Sara Cheleschi et al. (2017)	β-Catenin	Downregulate the expression of MMP-13, ADAMTS-5, and HDAC-4
miR-146a	Sara Cheleschi et al. (2017)	β-Catenin	Downregulate the expression of MMP-13, ADAMTS-5, and HDAC-4
miR-138	Xu Weiling et al. (2019)	β-Catenin	Alleviate OA cartilage severity through increasing the level of Col2a1 and aggrecan and reducing the level of MMP-13
miR-155-5p	Alessandra Colombini, et al. (2021)	β-Catenin	Protect the chondrocytes through reducing the level of MMP-1 and MMP-3 via inhibiting the Wnt signaling pathway
miR-34a	Sara Cheleschi, et al. (2020)	β-Catenin	Modulate the inflammation through regulating the expression of MMP-13 and ADAMTS-5
miR-10a	Li J. et al. (2015)	β-Catenin	Inhibited osteogenic differentiation and decreased mouse umbilical vein endothelial cell proliferation and migration
miR-410	Zhang Yanjie et al. (2017)	Wnt3a	Increase chondrogenic markers of Col2a1, Sox9, ACAN, and Has2, and alleviate OA cartilage severity
miR-497-5p	Hou Liying et al. (2019)	Wnt3a	Increase the expression of Col2a1 and aggrecan, and reduce the expression of MMP-13 and ADAMTS-4
miR-92a-3p	Mao Guping et al. (2018)	Wnt5a	Regulate cartilage development and homeostasis
miR-374a-3p	Shi Feng-Lei et al. (2020)	Wnt5b	Alleviate LPS-induced damage in CHON-001 cells and inhibit cartilage injury
miR-1246	Peng Sisi et al. (2021)	GSK3β and Axin2	Promote inflammation *via* increasing the level of IL-6, IL-8, MMP-3, and MMP-13
miR-140-3p	E. Ntoumou et al. (2017)	Wnt5a	Downregulate in the serum of OA patients and modulate the metabolic processes of OA pathology
miR-671-3p	E. Ntoumou et al. (2017)	Wnt5a	Downregulate in the serum of OA patients and modulate the metabolic processes of OA pathology
miR-33b-3p	E. Ntoumou et al. (2017)	Wnt5a	Downregulate in the serum of OA patients and modulate the metabolic processes of OA pathology
miR-520b	Alessandra Colombini et al. (2021)	DKK	Modulate inflammatory functions and activate the Wnt signaling pathway
miR-302d-3p	Alessandra Colombini et al. (2021)	DKK	Modulate inflammatory functions and activate the Wnt signaling pathway
miR-520c-3p	Alessandra Colombini et al. (2021)	DKK-1	Modulate inflammatory functions and activate the Wnt signaling pathway
miR-154-5p	Li Jianwei et al. (2015)	Wnt11	Inhibit osteogenic differentiation *via* decreasing the activity of the non-canonical Wnt/PCP (RhoA-ROCK) pathway
miR-26b	Sun Jilin et al. (2015)	Wnt	Inhibit the TNF-α, IL-1β, and IL-6 levels and reduce the proliferation of rheumatoid arthritis synovial fibroblasts
miR-335-5p	Zhang Jin et al. (2011)	DKK1	Inhibit the osteoblasts and hypertrophic chondrocytes of mouse embryos to promote osteogenic differentiation

As a key component of the Wnt signaling pathway, the activation of *β*-catenin depends on its transport and accumulation in the nucleus. Once it is in the nucleus, it can bind to TCF/LEF transcription factors to initiate gene transduction. Therefore, many scholars have studied the effect of miRNAs on *β*-catenin. Dong et al. (2021) found that miR-127-3p can protect chondrocytes from IL-1-induced damage by decreasing the expression of MMP-13, ADAMTS-5, TNF-α, and IL-6 by suppressing the activation of *β*-catenin. miR-138 has also been proven to have the same effect, alleviating OA cartilage severity by increasing the levels of Col2a1 and aggrecan and reducing MMP-13 levels by targeting the *β*-catenin gene (Xu et al., 2019). Cheleschi et al. (2017) investigated the effects of miRNAs in both normal and OA human chondrocytes in regulating the levels of MMP-13 and ADAMTS-4. The results showed that several miRNAs, such as miR-27a/b, miR-140, and miR-146a/b, are responsible for modulating the *β*-catenin level and the aforementioned changes in chondrocytes. Researchers found that miR-10a reduced *β*-catenin at both the protein and transcriptional levels and inhibited osteogenic differentiation to reduce cartilage damage in OA (Li et al., 2015), and other researchers found that the upregulation of *β*-catenin induced by miR-195-5p could promote the inflammatory activity and apoptosis rate of chondrocytes (Shu et al., 2019).

Wnt and its receptors can be targeted and modulated by miRNAs. MiR-410 expression is upregulated during the process of chondrogenic differentiation in MSCs and can increase the levels of Col2a1, Sox9, and ACAN by regulating the target gene Wnt3a (Zhang et al., 2017). Wnt3a is also a target gene for miR-497-5p, which regulates the activity of the Wnt signaling pathway in OA (Hou Liying et al., 2019). Indeed, elevated levels of miR-497-5p prominently increased Col2a1 and aggrecan expression and decreased the expression of the catabolic proteinases MMP-13 and ADAMTS-4, while Wnt3a overexpression reversed these effects (Hou Liying et al., 2019). Similarly, miR-92a-3p could reduce cartilage matrix synthesis by targeting Wnt5a and enhancing its expression (Mao et al., 2018). Ntoumou et al. (2017) investigated serum samples collected from healthy people and OA patients and found that miR-140-3p, miR-33b-3p, and miR-671-3p were downregulated in the serum of OA patients and modulated the metabolic processes of OA pathology by regulating the expression of the Wnt5a gene.

Many studies have reported that miRNAs could participate in the regulation of biological processes by regulating the expression level of antagonists of the Wnt signaling pathway. DKK-1 has been extensively studied, and it has been reported that miR-335-5p can downregulate DKK-1 and activate Wnt signaling to promote osteogenic differentiation in mice (Zhang et al., 2011). Colombini et al. (2021) studied the miRNA cargo embedded in extracellular vesicles (EVs) released from adipose-derived mesenchymal stromal cells. Intriguingly, they found that miR-520c-3p was upregulated, but miR-302d-3p was silenced, and both targeted DKK to activate Wnt signaling to modulate inflammatory functions.

### Wnt signaling in OA experimental animal models

Some studies have investigated the role of the Wnt signaling pathway in the articular pathologies of OA. Ryu and Chun (2006) studied the effect of IL-1 on cultured rabbit chondrocytes *in vitro*. They found that IL-1 could increase the expression of Wnt5a, which is strongly related to cartilage destruction, by inhibiting collagen-II in OA. Blom et al. (2009) studied the expression of the Wnt and Fzl genes in experimental OA and human OA. They found that Wnt and Wnt-related genes are highly expressed in the articular cartilage and synovium in experimental OA and human OA. Among these genes, the Wisp1 gene, which induces Wnt gene expression, is significantly highly expressed in chondrocytes. Secreted frizzled-related proteins (sFRPs) are antagonists of the Wnt pathway and play an important role in the Wnt pathway; sFRP (−/−) mice are more liable to develop OA because of the increased activity of MMPs (Lories et al., 2007; Thysen et al., 2015). These findings indicate revealed the effect of the Wnt/β-catenin signaling pathway in OA. Chen et al. (Chen et al., 2008; Zhu et al., 2008; Zhu et al., 2009) confirmed that an increased *β*-catenin protein level in the nucleus is accompanied by a reduction in *β*-catenin degradation in chondrocytes. These changes could result in an OA-like phenotype, including chondrocyte differentiation, increased MMP and Col10a expression, loss of articular cartilage, and osteophyte formation.

The ADAMTS family of proteases and matrix metalloproteinase (MMPs) are strongly involved in the cartilage degradation of OA (Glasson et al., 2005; Roach et al., 2005; Majumdar et al., 2007). The deletion of ADAMTS-5 or MMP-13 or double KO of both ADAMTS-4 and ADAMTS-5 prevented cartilage degradation in a surgically induced knee OA mouse model, while the expression of ADAMTS-4 and ADAMTS-5 was increased in *β*-catenin-overexpressing mice (Majumdar et al., 2007; Wang et al., 2013; Yang et al., 2015).

Several studies have found that the levels of MMP-3, MMP-9, MMP-13, and ADAMTS-5 were closely related to those of Wnt/β-catenin, and inhibiting the expression of *β*-catenin could reduce the expression of MMPs and ADAMTS-5 and reduce the damage to articular cartilage in an animal OA model (Bai et al., 2019; Li et al., 2019; Xie et al., 2019). These findings revealed that MMPs and ADAMTS4/5 affect cartilage destruction and the progression of OA through the Wnt/β-catenin pathway, and targeting this pathway can be a strategy for treating or preventing OA.

### Interventions in the Wnt cascade

The complexity of the Wnt signaling pathway means that regulating it is a complex and challenging process. Pharmaceutical interventions on the Wnt cascade have hitherto mainly focused on secreted Wnt antagonists. Because the mechanism of the Wnt signaling pathway requires a combination of Lrp, Fzl, and Wnt to initiate the downstream *β*-catenin molecule, their inhibitory actions on Wnt are different. They can be divided into endogenous Wnt pathway inhibitors and pharmaceutical inhibitors according to the mechanism of their action on the signaling pathway.

### Endogenous inhibitors of the Wnt signaling pathway

The Wnt pathway is controlled by several endogenous antagonists, and the decreased expression levels of certain antagonists may alleviate the progression of OA. Our current understanding of certain endogenous antagonists of the Wnt signaling pathway in OA, as discussed in the following sections, is summarized in Table 4.

**TABLE 4:** Endogenous inhibitors of the Wnt/β-catenin signaling pathway.

Author	Subjects	Inhibitor	Path involved	Results
Wafa Bouaziz et al. (2015)	Murine chondrocytes and mice with a DMM model	Sclerostin	Wnt3a/β-catenin	Sclerostin inhibited expression of ADAMTSs, MMPs, and Col10a by suppressing the canonical Wnt pathway and sclerostin through Wnt3a/β-catenin pathway
Hwanhee Oh et al. (2012)	C57BL/6 mice with a DMM model and articular chondrocytes	Dkk-1	DKK-1	Overexpression of Dkk1 by intraarticular injection of AdDkk-1 inhibited DMM-induced experimental OA, DKK-1 inhibited Wnt-3a-catenin-mediated upregulation of MMP-13 and ADAMTS-4
Diederik P C de Rooy et al. (2013)	Serum levels of RA patients	DKK-1	DKK-1	RA patients with risk alleles of genetic variants in Dkk-1 are inclined to joint destruction over time
S J B Snelling et al. (2016)	Human OA cartilage and synovial tissues, human chondrocytes	DKK-3	DKK-3	Dkk-3 is upregulated in OA and may have a protective effect on cartilage integrity by preventing proteoglycan loss
Nicole C Walsh et al. (2009)	C57BL/6 J mice	DKK-1, SFR1	DKK-1, SFR1	The Wnt signaling antagonists DKK1 and sFRP1 are expressed in inflamed synovial, suggesting that the inhibition of Wnt signaling contributes to impaired osteoblast function within arthritic bone tissues in RA
Shu-Guang Gao et al. (2016)	Human OA cartilage and subchondral bone from tibial plateau	WIF-1	WIF-1	Patients with disease had significantly decreased WIF-1 levels. Thus, WIF-1 levels are negatively correlated with the severity of the disease
Jean Cassuto et al. (2018)	Plasma derived from twenty-four osteoarthritis patients	Dkk-1, sFRP-1	Dkk-1, sFRP1	Dkk-1 and sFRP1 suppress osteogenic activation of MSCs and they are required for full osteoblastic differentiation

Sclerostin is an antagonist of the Wnt pathway and can antagonize the combination of Wnt with LRP and FZL. Sclerostin inhibits the expression levels of the downstream catabolic effector genes Runx-2, MMP-13, ADAMTS-4, and ADAMTS-5 by decreasing the mRNA expression levels of *β*-catenin in healthy chondrocytes, while sclerostin knockout mice exhibit increased expression of aggrecanases and Col10a (Lewiecki, 2014; Bouaziz et al., 2015; Wu et al., 2017). Dkk-1 is another Wnt antagonist, and its overexpression can reduce the degeneration of cartilage and OA in animal models induced by destabilization of the medial meniscus (DMM). Dkk-1 can inhibit Wnt-mediated expression of catabolic genes, including MMPs, Runx-2, and ADAMTS-4/5 (Oh et al., 2012; de Rooy et al., 2013; Funck-Brentano et al., 2014). Dkk-3 is another member of the Dkk family of Wnt antagonists, and it inhibits the loss of proteoglycan and collagen from cartilage in a model of OA (Snelling et al., 2016). A previous study indicated that the expression of Dkk-1 is related to inflammation and is consistent with the process of OA, while Dkk-2 and Dkk-3 expression is associated with late stages of the disease (Walsh et al., 2009). WNT-inhibitory factor 1 (WIF-1) is an extracellular Wnt antagonist that has been investigated in several studies. Bleil et al. (2015) found that WIF-1 could reduce the expression of *β*-catenin in the chondrocytes of OA, while other researchers found a similar tendency and confirmed that the WIF-1 level was negatively correlated with the severity of the disease in OA (Gao et al., 2016; Cassuto et al., 2018). The sFRP family, which has five members (SFRP1–5), was originally identified as an extracellular ligand-binding inhibitor of the Wnt signaling pathway (Thysen et al., 2015). More recent data indicate that SFRPs have a variety of physiological functions, including regulating cell metabolism and intracellular signaling by regulating or inhibiting the activity of the Wnt signaling pathway. The sFRP1 level strongly influences the synthesis of the cartilage matrix, and animal experiments showed that sFRP1(−/−) mice had greater subchondral bone plate thicknesses in a DMM model of OA (Thysen et al., 2015). sFRP3 also negatively regulated the key gene *β*-catenin in the Wnt signaling pathway in RA and decreased the expression of MMP-3 (Elhaj Mahmoud et al., 2017).

### Pharmaceutical inhibitors of the Wnt signaling pathway

Recently, a number of new and classic drugs were investigated in terms of their inhibitory effect on the Wnt pathway in OA. Most of them influence the pathological process of OA by modulating Wnt pathway activity, as summarized in Table 5. Zhong et al. (2018) found that artemisinin (ART) could inhibit the expression of proinflammatory chemokines and cytokines, including IL-1β, IL-6, TNF-α, and MMP-13, suppressing the degeneration of the articular cartilage. These results indicate that ART alleviates the IL-1β-mediated inflammatory response and OA progression by regulating the Wnt5a/β-catenin signaling pathway. Mianserin suppresses Rspo2-induced accumulation of *β*-catenin and phosphorylation of LRP6, which play an important role in preventing OA progression in a rat model of OA (Okura et al., 2019). Growth differentiation factor-5 (GDF-5) is strongly related to ECM homeostasis. It could inhibit the expression of MMP-13 and ADAMTS-4 and stimulate the expression of the cartilage anabolic genes Acan and Sox9 in human chondrocytes through intervention in the Wnt canonical signaling pathway by the overexpression of Dkk-1 and FRZB (Enochson et al., 2014). Ma et al. (2019) demonstrated that a systemic injection of rapamycin could reduce the degeneration of cartilage by inhibiting *β*-catenin in an animal model of OA. The mechanism by which rapamycin attenuates OA mainly relies on a reduction of apoptosis and activation of autophagy in chondrocytes. Icariin exerts a chondroprotective effect by inhibiting the expression of *β*-catenin *in vivo* and *in vitro*, and it may have therapeutic potential for the treatment of OA (Zeng et al., 2014). Several researchers have found that the expression of some large molecules, such as FYN and high-molecular-weight fibroblast growth factor-2 (FGF-2), is elevated and correlated with the expression of Wnt/β-catenin in human OA cartilage and mouse models. Inhibiting these molecules can reduce the levels of the extracellular matrix catabolic enzymes and delay articular cartilage degeneration by blocking the *β*-catenin pathway (Zhao et al., 2015; Li et al., 2018; Meo Burt et al., 2018).

**TABLE 5:** Pharmacological inhibitors of the Wnt/β-catenin signaling pathway.

Author	Subjects	Inhibitor	Path involved	Results
Zhong Gang et al. (2018)	Human OA chondrocytes and rats OA model	Artemisinin	Wnt5a/β-catenin	ART inhibits OA progression and cartilage degradation by exhibiting potent anti-inflammatory effects *via* the Wnt/β-catenin signaling pathway
L Enochson et al. (2014)	Human OA chondrocytes	Growth differentiation factor 5 (GDF5)	Wnt/β-catenin	GDF5 inhibits expression of MMP-13 and ADAMTS-4 and stimulates the expression of cartilage anabolic genes ACAN and SOX9 *via* the DKK1-mediated Wnt signaling pathway
Long Ma et al. (2019)	C57BL/6 mice	Rapamycin	Wnt/β-catenin	Rapamycin injection could activate chondrocyte autophagy, increase the expression of LC3 and ATG-5, reduce OARSI scores, expression of *β*-catenin, MMP-13, and chondrocyte apoptosis
Li Zeng et al. (2014)	Human SW 1353 cells and rats OA model	Icariin	Wnt/β-catenin	Icariin could decrease the MMP-13 expression and reduce the number of cartilage lesions *via* the Wnt/catenin pathway
Yun-Peng Zhao et al. (2014)	C57/BL6 mice and chondrocytes	Progranulin	Wnt/β-catenin	Progranulin suppresses inflammatory action of TNF-α and inhibits the activation of *β*-catenin signaling in chondrocytes, protects cartilage degradation in spontaneous and surgically induced OA models
Caressa Lietman et al. (2018)	Human chondrocytes and C57BL/6 J mice with DMM method of OA model	XAV939	XAV939	XAV-939 ameliorates OA severity associated with reduced cartilage degeneration and synovitis
Jingyuan Li et al. (2019)	Sprague–Dawley rats and ATDC5 cells	Xanthan gum	Wnt3a/β-catenin	Xanthan Gum reduces the OARSI score and the concentration of inflammatory cytokines in OA. Xanthan Gum acted on Wnt3a/β-catenin in ATDC5 cells to decrease the expression of MMP-13 and ADAMTS-5
Yuzhe He et al. (2019)	Rat chondrocytes and an OA rat model	Costunolide	Wnt/β-catenin	Costunolide decreases IL-1β-induced upregulation of MMPs, INOS, COX-2 and IL-6, increases the expression of Clo2a1 and Sox9 through inhibiting Wnt/β-catenin signaling pathway
Fuen Liu et al. (2020)	C57BL/6 mice and human chondrocytes derived from patients	Scutellarin	Wnt3a/β-catenin and MAPK signaling pathway	Scutellarin downregulates the mRNA and protein expression of MMP-1, MMP-13, and ADAMTS-5 and promotes the expression of Col2a1 and aggrecan. Moreover, scutellarin inhibits the migration of *β*-catenin and phosphorylation of p38 into the nucleus
J.-B. Gao et al. (2020)	Human chondrocytes and human knee OA cartilage tissue	Fibulin-5	Wnt/β-catenin	Fibulin-5 increases the expressions of Col2a1 and aggrecan and reduces IL-1β-induced inflammation of chondrocytes, as well as expressions of IL-6, IL-8, and TNF-αvia reducing the activity of Wnt/β-catenin signaling pathway
Qian-Hai Ding et al. (2018)	Rat articular cartilage and chondrocytes	Emodin	NF-κB and Wnt/β-catenin signaling	Emodin dose-dependently down-regulates the expression of MMP-3, MMP-13, ADAMTS-4 and ADAMTS-5 at both the mRNA and protein level in IL-1β-stimulated rat chondrocytes. In addition, the IL-1β-induces activation of NF-κB and Wnt signals was attenuated by emodin
Shan Cong et al. (2021)	Sprague–Dawley (SD) rats	Iguratimod	β-Catenin	Iguratimod improves the degeneration of articular cartilage and decreases the levels of MMP-13, TNF-α, and IL-6 in serum. Iguratimod downregulates the mRNA and protein expression of *β*-catenin *in vivo*
Xindie Zhou et al. (2016)	New Zealand rabbits and chondrocytes	Palmatine	Wnt/β-catenin and Hedgehog signaling pathways	Palmatine decreases the expression of the MMPs and increases the synthesis of TIMP-1, whereas collagenase II and aggrecan are inhibited by IL-1β. Palmatine protects the cartilage degradation *via* suppressing the Wnt and hedgehog signaling pathways
Yang Xi et al. (2020)	Sprague–Dawley rats and chondrocytes	Erdosteine	MAPK, NF-κB, and Wnt/β-catenin signaling pathways	Erdosteine suppresses the expressions of IL-1β-induced production of inflammatory factors COX-2 and iNOS and attenuates the degradation of ECM by repressing the expression of MMP-1, MMP-3, and MMP-13. Moreover, erdosteine could inhibit the activation of IL-1β-induced MAPK and Wnt/β-catenin
Yi-Yue Chen et al. (2019)	Human OA, normal articular cartilage samples, and chondrocytes	Cyclin D1	Wnt3/β-catenin	Cyclin D1 inhibits cell apoptosis and cell cycle promotes the proliferation OA chondrocytes through activating the Wnt/β-catenin signaling pathway
Tangbo Yuan et al. (2022)	107 OA patients	Curcumin	Wnt/β-catenin	Curcumin can effectively decrease the pathological results of OA, with a remarkable safety profile; its mechanism may be the activation of the Wnt/β-catenin signaling pathway to inhibit the inflammatory reaction and apoptosis in chondrocytes
Long Ma et al. (2021)	Female C57BL/6 J OA model mice with ACLT + DHA method	Dihydroartemisinin	Wnt/β-catenin	Dihydroartemisinin decreases MMP-13 and VEGF expression in the articular cartilage, and finally results in decreasing OARSI scores and reducing articular cartilage degeneration. In addition, dihydroartemisinin reduces abnormal subchondral bone remodeling *via* the Wnt/catenin
Xindie Zhou et al. (2013)	New Zealand rabbits and chondrocytes	Tetrandrine	Wnt/β-catenin	Tetrandrine decreases the expression of MMP-1, MMP-3, MMP-13, TIMP-1, and *β*-catenin in rabbit chondrocytes and cartilage
De-Heng Chen et al. (2021)	Human chondrocyte and C57BL/6 J OA model	Oroxylin A	NF-κB and Wnt/β-catenin signaling	Oroxylin A could rescue IL-1β-mediated hypertrophic alterations of chondrocytes and inhibit the ECM hemostasis in human chondrocytes. Oroxylin A attenuates the IL-1β-induced hypertrophic changes in chondrocytes by inhibiting the Wnt/β-catenin signaling pathway
Nan Yao et al. (2021)	Sprague–Dawley rats	Bushen Qiangjin capsule	Wnt/β-catenin	Bushen Qiangjin capsule obviously reduces pathological damage and matrix degradation of articular cartilage in KOA rats and downregulates mRNA and protein expression of Wnt-4, *β*-catenin, Frizzled-2, and caspase-3
Wei-Ping Chen et al. (2017)	Rat chondrocytes	Licochalcone A	NF-κB and Wnt/β-catenin signaling pathways	Licochalcone A inhibits ADAMTS-5, ADAMTS-4, MMP-13, and MMP-1 expression *via* inhibiting the NF-κB and Wnt/β-catenin signaling pathways in rat chondrocytes
Tao Yang et al. (2020)	Rat chondrocytes	Vitamin D3	Wnt3a/β-catenin	Vitamin D3 and PNU-74654 could attenuate the effects induced by TNF-α and increase the level of col2a1 and aggrecan and decrease the expression of MMP-3 and MMP-13, ADAMTS-4, ADAMTS-5, Wnt-3a, and nuclear *β*-catenin
V Deshmukh et al. (2018)	SW480 cells and Sprague–Dawley rats	SM04690	Wnt/β-catenin	SM04690 induces hMSC differentiation into chondrocytes, decreases cartilage catabolic marker levels, promotes cartilage growth, and improves joint health in a rat model of knee OA
Hanting Xia et al. (2020)	MIA-induced OA rat models and chondrocytes	Jiawei Yanghe decoction (JWYHD)	Wnt/β-catenin	JWYHD increases the chondrocyte viability against IL-1β-induced chondrocyte apoptosis and preserves glycosaminoglycans in the extracellular matrix. JWYHD promotes chondrocyte viability against apoptosis and decreases MMP-3, MMP-13, caspase-3, and caspase-9 *via* the Wnt/β-catenin signaling pathway

## Conclusion

In recent years, there has been growing interest in trying to identify the mechanisms underlying pathological changes in OA for the purpose of developing a targeted treatment strategy for OA. Extensive studies of the Wnt signaling pathway have revealed multiple functions of the Wnt signaling cascade in the maintenance of joint homeostasis, particularly in cartilage. The Wnt cascade should be tightly regulated because either overexpression of Wnt signaling or blockade of its activation may result in cartilage damage and bone erosion. The Wnt signaling pathway involves several levels, including the extracellular, intracytoplasmic, and nuclear levels, so regulation should exist in multiple aspects. New strategies to target and control these different levels of regulatory mechanisms may lead to the preservation of cartilage or the healing of diseased joints. It is absolutely necessary to investigate the Wnt signaling pathway in greater detail, not only to develop specific therapeutic interventions that target this pathway but also to discover and utilize new biomarkers for personalizing medical approaches in the future.

## Glossary

Acan: Aggrecan
ADAMTS: a disintegrin and metalloproteinase with thrombospondin motifs
ACLT: anterior cruciate ligament transection
ART: artemisinin
APC: adenomatous polyposis *coli*
CaMKII: calcium/calmodulin-dependent kinase II, calcium/calmodulin-dependent protein kinase type II
CK-I: casein kinase I
Col2a1: type II collagen alpha 1 chain
Dkk: dickkopf
Dvl: disheveled
DMM: destabilization of the medial meniscus
ECM: extracellular matrix
EVs: extracellular vesicles
FGF-2: fibroblast growth factor-2
GSK-3β: glycogen synthase kinase-3 *β*
IL: interleukin
JNK: c-Jun N-terminal kinase
LRP: lipoprotein receptor-related protein
mRNA: messenger RNA
miRNA: microRNA
MMP-13: matrix metalloproteinase 13
MMx: medial meniscus resection
OA: osteoarthritis
PKC: protein kinase C
PLC: phospholipase C
Runx-2: Runt-related transcription factor-2
Ryk: receptor tyrosin kinase
sFRPs: secreted frizzled-related proteins
Sox9: SRY-box transcription factor 9
TNF-α: tumor necrosis factor alpha
Wnt: Wingless/integrated
WIF-1: WNT-inhibitory factor-1
